# Three Cases of Uterine Fibroid Tumors Treated by Ergotine

**Published:** 1876-07

**Authors:** D. A. K. Steele

**Affiliations:** Assistant Surgeon


### REPORT OF THREE CASES OF UTERINE FIBROID TUMORS TREATED BY ERGOTINE,

### AT THE DISPENSARY OF THE WOMAN'S HOSPITAL OF THE STATE OF ILLINOIS.

By D. A. K. STEELE, M.D., Assistant Surgeon.

(Read before the Chicago Society of Physicians and Surgeons, March 27, 1876.)

Simultaneously with the publication of Hildebrandt's articles on the curative influence of ergotine upon fibroid tumors of the uterus, a general investigation was entered upon by the medical world to determine by practical experiment the influence exerted by this drug over a hitherto almost incurable and often undiagnosed disease. The only method of determining its clinical value, is by a careful synopsis of cases treated—not the successful ones alone, but all as they fall under the reporter's observation.. The following report is made, not for its intrinsic value or for the development of any new features, but simply as an addition to the literature of this important field of observation. The cases are from notes by the House Physician, Dr. Harriet M. Kollock.

Case I. Mrs. M. N., (colored) American, aged 34, presented herself for treatment at the dispensary, March 21st, 1875, giving the following history: Married at 14; menstrual periods always regular until the past year. Has no children, but had two miscarriages eight or nine years ago. She thinks they occurred about the third or fourth months of pregnancy, and were induced by over work. One year ago she had a severe attack of sickness and was confined to her bed for six weeks, suffering from extreme pain through the uterus and lower part of abdomen. Her physician called it "inflammation of the womb." Since that time her menstrual periods have been a little more profuse and have occurred more frequently, until now there is an interval of but two weeks or less, and they continue nine or ten days. Formerly lasted but three or four days, with a month's interval. She complains of severe pain through the pelvis, especially in the left side, which is increased by walking or standing any length of time.

Examination revealed a fibroid growth in the left and posterior portion of uterine wall that could be distinctly outlined through the rectum and the left abdominal wall. Cervix hard and nodular; depth of womb three and one-fourth inches.

Treatment advised, was daily hypodermic injections of twelve drops Squibb's fl. ext. ergotine.

This treatment was continued for four or five days, but as each injection produced considerable local disturbance and there were evidences of abscesses forming, both on arms and abdomen, where injections had been inserted, it was deemed best to discontinue this mode of administration, and the patient was directed to take ten drops twice daily, internally. She complained of having strong uterine contractions after each injection, lasting from half an hour to one hour, causing her considerable pain. The same symptoms were felt after each dose taken internally, but in a less degree.

April 14th. Patient has now taken the ergotine steadily for twenty-four days; general health much improved; has had one menstrual period during that time, continuing five days, after an interval of eighteen days. On examination the tumor appeared notably decreased in size.

June 15th. She reported at the dispensary, having taken the ergotine regularly since the 21st of March, (about three months); continued improvement in health. Periods occur at intervals of about 24 days and continue five or six, less profuse than before.

Examination. Tumor about in the same condition as when examined April 14th. Ergotine was then discontinued.

Case II. Mrs. E. G., American, æt. 31, applied for treatment June 5th, 1875, stating that she began menstruating at the age of 13; had one period, when she took cold and had no more for six months; with this exception, was regular until she married, seven years ago. Since then, the periods have occurred more frequently, lasting from five to ten days. The intervals are never longer than three weeks, and any excitement or overwork will bring on a profuse flow, which is usually accompanied by severe pain and the expulsion of large clots. Had a constant leucorrhœa two years ago, which was improved by local treatment. Now it occurs only just before and after menstruation. Complains of pain in the left ovarian region, in the left breast, and head; severe backache and palpitation of the heart; bowels constipated; appetite poor; menses frequent and painful. Was treated two years ago for tumor, at which time the abdomen was much enlarged; general decline of health for past two years.

On examination find a fibroid tumor occupying the left anterior uterine wall. It can be distinctly outlined through the abdominal walls, the fundus of uterus can be felt about midway between the pubes and umbilicus. Sound enters womb three and one-fourth inches.

June 7th. Commenced treatment by injecting in the arm, near insertion of the deltoid, twelve drops of Squibb's fl. ext. ergotine; this was continued daily until July 28th. The injections gave rise to no local irritation, but caused the patient to complain of severe uterine contractions and increased pain in the left breast, continuing about an hour after each injection.

June 30th. Patient reported her general health much improved. Her menstrual period continued but four days, and the interval since the last period was increased by five days.

Examination shows little or no difference in the size of the growth.

July 28th. Patient again examined. Tumor about the same size as when she applied for treatment; general health decidedly improved; menstrual periods occur now about once a month and are not at all profuse.

The ergotine was discontinued.

Jan. 7th, 1876. Patient has had no treatment since July 28th (six months.) Periods have been regular, painless and not profuse during that time.

Examination shows the tumor to be gradually increasing in size.

Case III. Mrs. R. L., American, æt. 19.

Aug. 4th, 1875. Has been married eighteen months; was always regular and well until prematurely confined of a six months child, one year ago. She got up six days after confinement. Had no menstrual period for three months, then had a slight show which gradually increased until for the last six months the flow has been constant, soiling three napkins daily; it is sometimes mixed with a leucorrhœal discharge. Complains of a constant pain through the uterine and ovarian regions; appetite good; bowels constipated.

Examination reveals ulceration of the cervix and a fibroid tumor in the anterior wall of the uterus; sound passed to the depth of three and a half inches. The ulceration was treated by an application of strong nitric acid, repeated again after an interval of two weeks, which entirely cured it.

Twelve drops of fl. ext. ergotine were hypodermically injected daily until Sept. 26th, when the tumor was found to have almost entirely disappeared. The hæmorrhage ceased entirely, after the first injection of ergotine, her menstrual period occurring the latter part of August, and again in September after a month's interval, and lasting but four days each time; all pain had vanished and her health much improved. The injections were discontinued and the patient advised to take twelve drops of the ergotine twice daily for a couple of weeks, and then return for a subsequent examination.

Oct. 23rd. Patient discharged, cured, the tumor having entirely disappeared, leaving nothing but a slight thickening of the anterior uterine wall. Depth of uterus two and a half inches.

Dec. 18th. Patient again examined. Uterus in a healthy condition; menstrual periods regular; general health good.

